# Monomeric Tartrate Resistant Acid Phosphatase Induces Insulin Sensitive Obesity

**DOI:** 10.1371/journal.pone.0001713

**Published:** 2008-03-05

**Authors:** Pernilla Lång, Vanessa van Harmelen, Mikael Rydén, Maria Kaaman, Paolo Parini, Claes Carneheim, A. Ian Cassady, David A. Hume, Göran Andersson, Peter Arner

**Affiliations:** 1 Division of Pathology, Department of Laboratory Medicine, Karolinska Institutet, Karolinska University Hospital, Huddinge, Sweden; 2 Department of Medicine, Karolinska Institutet, Karolinska University Hospital, Huddinge, Sweden; 3 Division of Clinical Chemistry, Department of Laboratory Medicine, Karolinska Institutet, Karolinska University Hospital, Huddinge, Sweden; 4 Integrative Pharmacology, Biovitrum AB, Stockholm, Sweden; 5 Institute for Molecular Bioscience, The University of Queensland, St Lucia, Australia; 6 The Roslin Institute and Royal (Dick) School of Veterinary Studies, University of Edinburgh, Roslin, United Kingdom; University of Parma, Italy

## Abstract

**Background:**

Obesity is associated with macrophage infiltration of adipose tissue, which may link adipose inflammation to insulin resistance. However, the impact of inflammatory cells in the pathophysiology of obesity remains unclear. Tartrate resistant acid phosphatase (TRAP) is an enzyme expressed by subsets of macrophages and osteoclasts that exists either as an enzymatically inactive monomer or as an active, proteolytically processed dimer.

**Principal Findings:**

Using mice over expressing TRAP, we show that over-expression of monomeric, but not the dimeric form in adipose tissue leads to early onset spontaneous hyperplastic obesity i.e. many small fat cells. In vitro, recombinant monomeric, but not proteolytically processed TRAP induced proliferation and differentiation of mouse and human adipocyte precursor cells. In humans, monomeric TRAP was highly expressed in the adipose tissue of obese individuals. In both the mouse model and in the obese humans the source of TRAP in adipose tissue was macrophages. In addition, the obese TRAP over expressing mice exhibited signs of a low-grade inflammatory reaction in adipose tissue without evidence of abnormal adipocyte lipolysis, lipogenesis or insulin sensitivity.

**Conclusion:**

Monomeric TRAP, most likely secreted from adipose tissue macrophages, induces hyperplastic obesity with normal adipocyte lipid metabolism and insulin sensitivity.

## Introduction

Adipocytes develop through a chain of events starting with the proliferation of mesenchymal stem cells (MSC) which subsequently differentiate into pre-adipocytes and finally into mature adipocytes [1]. The exact mechanisms regulating these transitions are not completely understood. Among transcription factors, peroxisome proliferator-activated receptor gamma (PPARγ) promotes adipocyte differentiation [2], while GATA-2, GATA-3 and Wnt inhibit [3], [4] this process. Human adipose tissue-derived MSCs can develop into multiple lineages including adipocytes, osteoblasts, chondrocytes and myocytes [5]. Conversely, osteoblasts and adipocytes are able to trans-differentiate [6]–[8], underscoring the close relationship between these cell types. One possible regulator of osteoblast differentiation and bone formation is the osteoclast-/macrophage-derived metalloenzyme tartrate-resistant acid phosphatase (TRAP) [9], [10].

TRAP, also known as purple acid phosphatase, uteroferrin or type 5 acid phosphatase (Acp5), exists either as a latent monomeric pro-enzyme of approximately 35 kDa or as a proteolytically processed two-subunit enzyme of approximately 22 and 16 kDa linked by a disulphide bridge [11], [12]. The proteolytic processing (exerted by, for instance, cathepsin K) excises part of an exposed loop region close to the active site and is permissive for the catalytic activation of TRAP [13]. TRAP has been shown to exist intracellularly in various cell types and to be secreted in vivo by osteoclasts [14], [15] and in vitro by macrophages and osteoclasts [16]. Intracellularly, TRAP is thought to participate in degradation of collagen fragments in osteoclasts [17], [18] and of phagocytosed bacteria in macrophages [19]. Osteoclast secreted extracellular TRAP, on the other hand, has been proposed to participate in the regulation of osteoclast adhesion and migration [20]. However, the role of extracellular TRAP secreted from other cell types, including macrophages, is unknown.

Macrophages are known to infiltrate adipose tissue during development of obesity [21], [22] and have been attributed a function in the link behind obesity and insulin resistance [22], [23]. In adipose tissue, macrophages have been proposed to secrete factors that increases expression of inflammatory genes in adipocytes [23], [24] and inhibit adipogenesis [24], [25]. Furthermore, disruption of PPARγ expression in macrophages predisposes mice to development of obesity and insulin resistance [26].

A clue to the link between macrophage infiltration of adipose tissue and disordered adipocyte function came from the unexpected biology of a TRAP over-expressing mouse transgenic line. To determine whether TRAP activity is limiting for bone turnover, a transgenic line was produced in which multiple additional copies of the complete gene were inserted in the germ line. The mice showed increased bone turnover, and over-expressed TRAP in the same locations as the endogenous genes [10]. The line was originally generated on a complex mixed genetic background. In the course of subsequent breeding and backcrossing to the FVB/N background, a sub-line of the transgenic mice was unexpectedly found to develop obesity. We therefore investigated the phenotype of the obese and a lean sub-line and also if TRAP can influence the proliferation and/or differentiation of adipocyte precursor cells. In this study, we present evidence that the obese phenotype in these animals is causally linked to over-production of TRAP. We demonstrate that certain macrophages in adipose tissue secrete monomeric TRAP that induces insulin-sensitive obesity by formation of new small adipocytes i.e. hyperplastic obesity. The results provide further evidence of the complex multifunctional roles of TRAP.

## Results

### Transgenic over expression of monomeric, but not proteolytically processed TRAP is associated with early onset spontaneous hyperplastic obesity in mice

**Figure 1:**
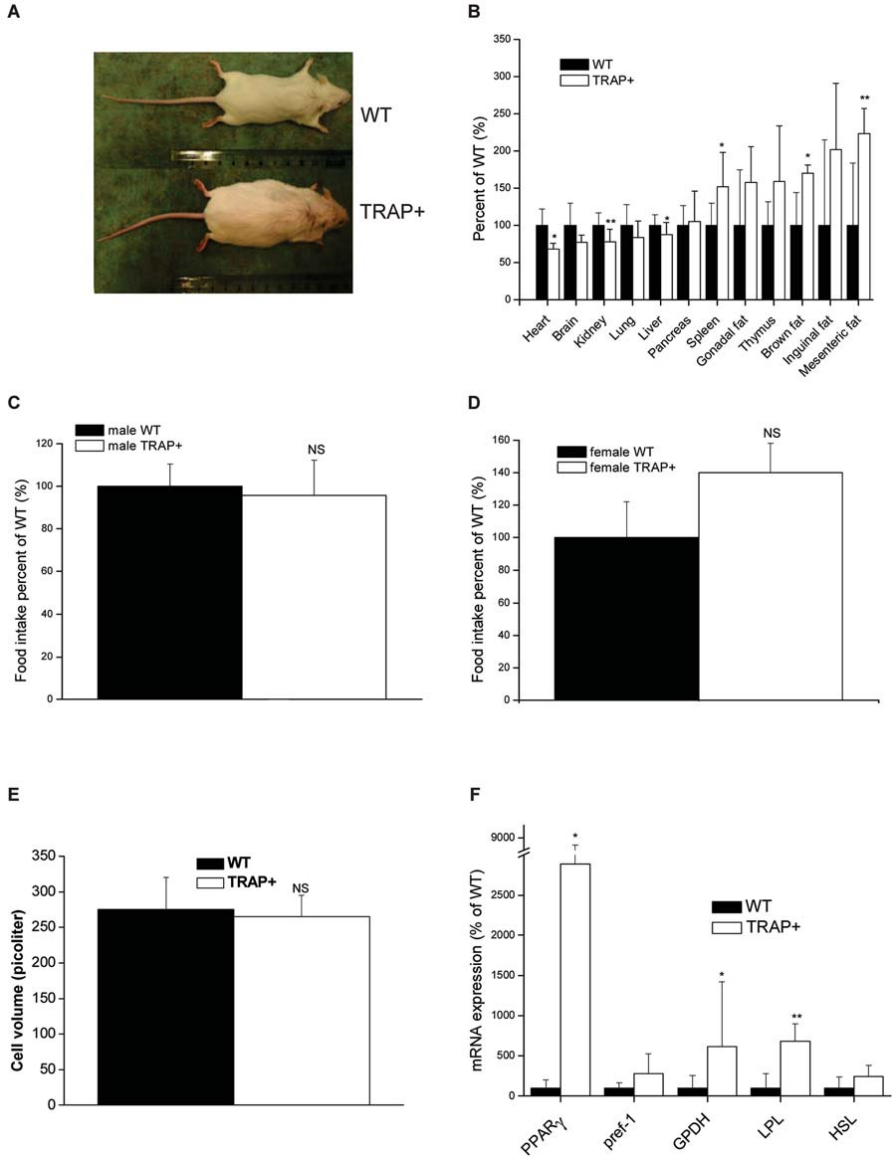
A transgenic TRAP over expressing mouse subline displays hyperplastic obesity. (A) Photograph of lean WT and obese TRAP+ mice. (B) Relative organ mass in TRAP+ compared to WT mice. (C) Food intake in male WT and TRAP+ mice. (D) Food intake in female WT and TRAP+ mice. (E) Adipocyte volume in WT and TRAP+ mice. (F) mRNA expression of adipocyte genes in adipose tissue from WT and TRAP+ mice.

During breeding of a number of TRAP over expressing mouse sub-lines derived from one of the original high-expressing founders [10], we noted that one of these sub-lines (TRAP+) gained excessive body weight after weaning and conspicuously developed spontaneous obesity (Figure 1A). The breeding of these lines was originally undertaken in an attempt to identify a more penetrant bone phenotype through association with other variables that affects bone density. Comparison of organ weights in TRAP+ versus non-transgenic littermate WT mice showed that two groups of organs were increased in TRAP+ mice; (1) lymphoid organs i.e. spleen by ∼50% and (2) different adipose tissue depots i.e. mesenteric- and brown fat by ∼60–120% depending on depot (Figure 1B and Table S2). On the other hand, the weights of other major internal organs e.g. liver, heart and kidneys, relative to total body mass were reduced in TRAP+ compared to WT mice. This increase in adipose tissue depots in TRAP+ mice was neither associated with increased food intake in adult males or females (Figure 1 C, D) nor increased adipocyte cell volume (Figure 1E). However, mRNA levels for several genes known to be associated with increased adipogenesis e.g. PPARγ (Z = 2.03 p = 0.042), glycerol-3-phosphate dehydrogenase (GPDH; Z = 2.13 p = 0.033) and lipoprotein lipase (LPL; Z = 2.68 p = 0.01) were significantly up-regulated in adipose tissue from TRAP+ compared to WT mice (Figure 1F).

**Figure 2:**
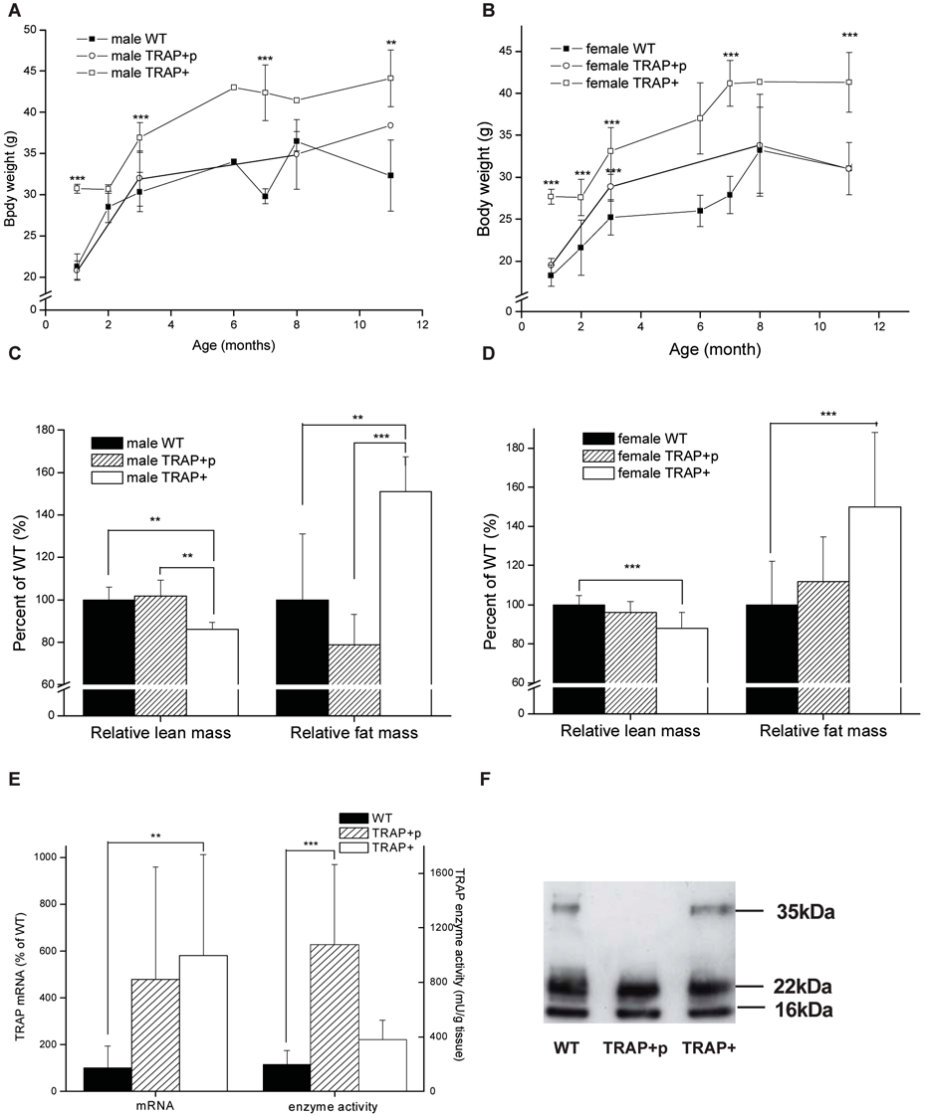
Over expression of monomeric TRAP is associated with obesity in transgenic mice. (A) Growth curve of male obese TRAP+ (open square), lean TRAP+p (open circle) and WT (black square) mice. (B) Growth curve of female obese TRAP+ (open square), lean TRAP+p (open circle) and WT (black square) mice. (C) Relative lean and fat mass in male WT, TRAP+p and TRAP+ mice. (D) Relative lean and fat mass in female WT, TRAP+p and TRAP+ mice. (E) Expression of TRAP mRNA and enzyme activity in adipose tissue from WT, TRAP+p and TRAP+ mice. (F) Western blot of TRAP in adipose tissue from WT, TRAP+p and TRAP+ mice.

The obese TRAP over expressing mouse sub-line (TRAP+) was next compared to an apparently lean transgenic sub-line from the same founder (TRAP+p). Comparison of growth curves of male and female WT, TRAP+p and TRAP+ mice showed that TRAP+ mice weighed 20–40% more than either the non-transgenic WT mice, or the lean transgenic TRAP+p mice (Figure 2 A, B and Table S3) throughout the first year of life. In male and female TRAP+ mice relative fat content was increased ∼50% compared to WT mice while lean body mass was decreased by ∼12–14% (Figure 2 C, D and Table S4). Neither relative lean nor fat mass differed in male or female TRAP+p mice compared to WT mice. Therefore, the obesity of the TRAP+ mice can be directly attributed to the TRAP transgene, which appears necessary but not sufficient to generate the phenotype.

The obese and lean TRAP over-expressing lines both expressed elevated levels of TRAP mRNA (WT vs TRAP+ Z = 2.94 p = 0.003) in adipose tissue (Figure 2 E), so the basis of differential adiposity is not due to differential expression of the transgene. The difference between the sub-lines emerged when the proteins were examined (Figure 2 F). The lean TRAP+p mice mainly expressed proteolytically processed TRAP with increased enzyme activity (WT vs TRAP+p Z = 3.80 p = 0.000143) (Figure 2E), whereas the obese TRAP+ mice expressed both monomeric and proteolytically processed TRAP in the adipose tissue (Figure 2F). We concluded therefore that the lean mice more effectively cleave TRAP, so that the obese mice, in effect, selectively over-express the monomeric form of the protein with low enzyme activity.

### Increased expression of monomeric, but not proteolytically processed TRAP in adipose tissue from obese humans

**Figure 3:**
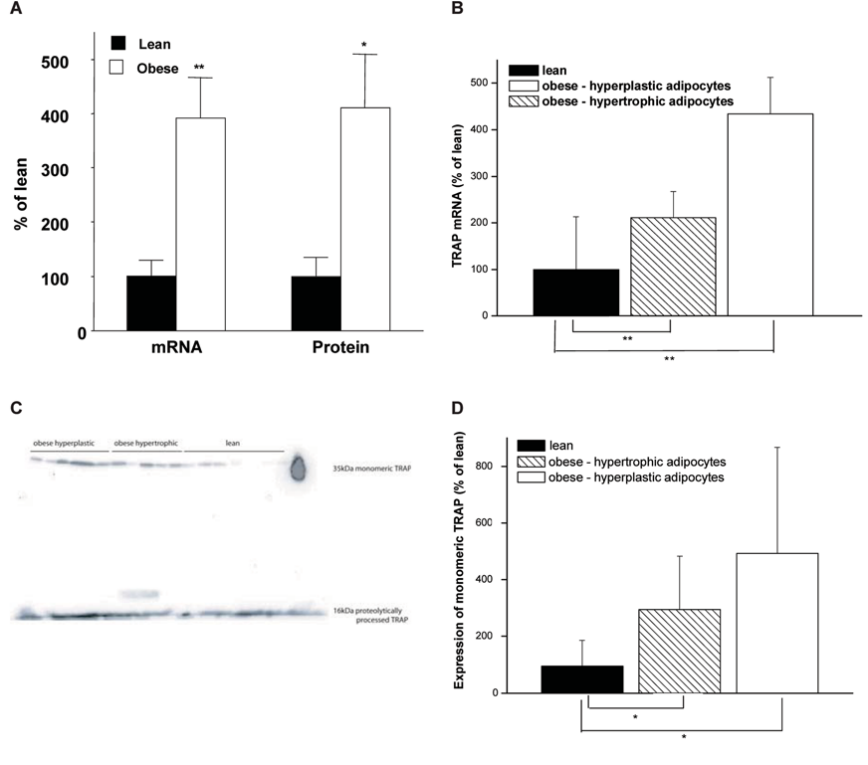
Expression of TRAP mRNA and protein in human adipose tissue. (A) Expression of TRAP mRNA and monomeric TRAP protein in obesity compared to the lean state. (B) TRAP mRNA, (C) total TRAP protein and (D) monomeric TRAP protein expression in hyperplastic and hypertrophic obesity.

Based upon the observations in mice, we asked whether monomeric TRAP is expressed in human adipose tissue, and whether it is over-expressed in obese patients. Both TRAP mRNA expression and monomeric TRAP protein expression were increased by 400% among the obese subjects compared to lean subjects (Figure 3A and Table S5). We also stratified the obese patient group into one group displaying a mainly hypertrophic phenotype (large fat cells) and one group displaying a more pronounced hyperplastic phenotype (small fat cells) in spite of similar body mass index. Although TRAP mRNA and monomeric TRAP protein were elevated in both obese groups, (Figure 3 B–D and Table S5), expression of proteolytically processed TRAP appeared not to be different (Figure 3C).

### Monomeric, but not proteolytically processed TRAP induces adipogenesis ex vivo in adipocyte precursor cells

To determine whether expression of monomeric TRAP in adipose tissue might have a direct causal role in generating obesity, we used in vitro assays. Adipocyte progenitor cells were simultaneously cultured under three different conditions; (1) control (standard medium), (2) in the presence of monomeric TRAP or (3) in the presence of proteolytically processed (cleaved) TRAP. For assessing effects on proliferation, the TRAP isoforms were present for 3 days in sub confluent, proliferating cultures. In differentiating cultures, the TRAP isoforms were added to sub confluent cultures for 2–3 days until confluence, as well as during induction of adipocyte differentiation for an additional 4 days.

**Figure 4:**
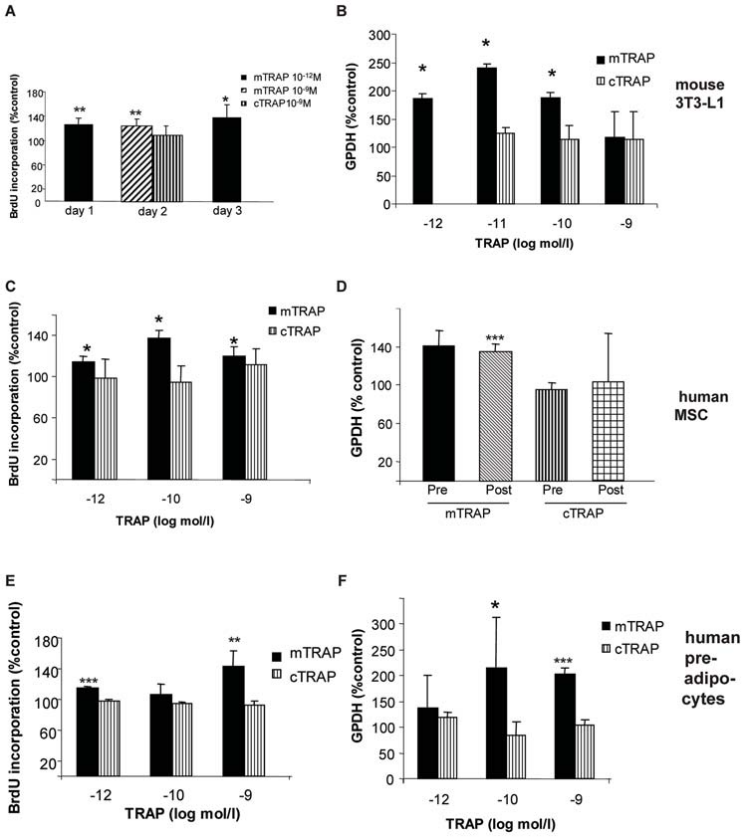
Stimulation of adipocyte proliferation and differentiation by monomeric TRAP but not proteolytically processed TRAP. The following cells were used: Mouse 3T3-L1 preadipocytes (A, B), Human mesenchymal stem cells (hMSC) (C, D) and human pre-adipocytes (E, F). Proliferation (BrdU) was assessed after 1 day (3T3-L1), 2 days (3T3-L1, hMSC), 3 days (3T3-L1) or 5 days (pre-adipocytes) after seeding. Differentiation (GPDH-activity) was measured 4 days (3T3-L1) or 12 days (hMSC, pre-adipocytes) after start of differentiation. hMSC were treated with TRAP either before (Pro) or at (Post) confluence. mTRAP = monomeric TRAP, cTRAP = proteolytically processed TRAP. Data are expressed as percentage of control. Statistical analysis was performed using T-test, * p<0.05, comparing control cells with stimulated cells.

Monomeric TRAP, at 10^−11^–10^−12^ M, caused a 30% enhancement of cell proliferation (Figure 4A and Table S6) and a 250% increase of terminal adipocyte differentiation (Figure 4B) in the mouse pre-adipocyte 3T3-L1 cell line. In contrast, proteolytically processed TRAP affected neither proliferation nor differentiation of this cell line (Figure 4A–B) in comparison to control cells. Similar differential effects of monomeric vs. proteolytically processed TRAP were also observed in adipocytes derived from human MSCs (Figure 4C–D) as well as human pre-adipocytes (Figure 4E–F), although at somewhat higher concentrations of monomeric TRAP. The stimulation of differentiation by monomeric TRAP was independent of the presence (Figure 4A, C, and E) or absence (data not shown) of the PPARγ activator roziglitazone.

### Macrophages are a source of monomeric TRAP in adipose tissue

**Figure 5:**
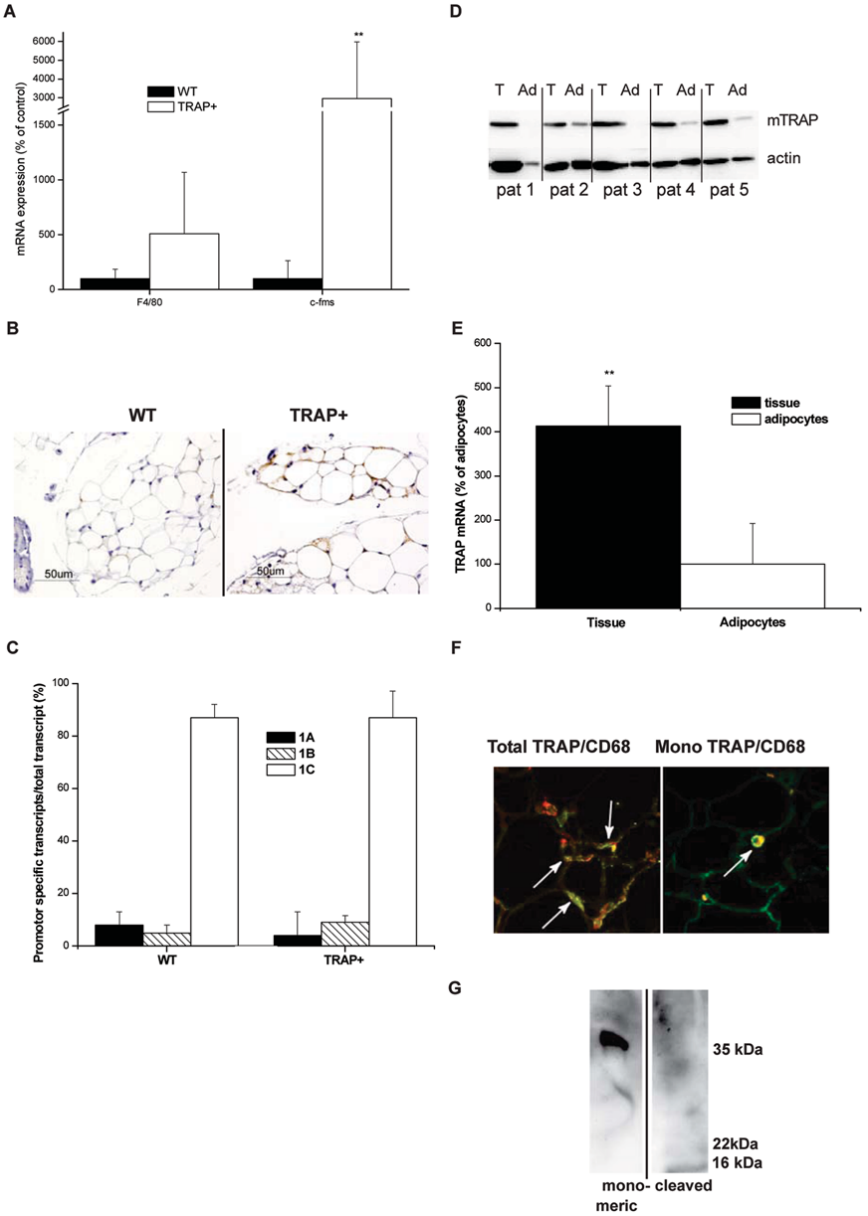
Macrophages are the primary source of monomeric TRAP in mouse and human adipose tissue. (A) mRNA expression of the monocyte/macrophage marker F4/80 and *c-fms* in adipose tissue of TRAP+ vs. WT mice. (B) Immunohistochemistry of mouse macrophage marker F4/80 in adipose tissue from TRAP+ compared to WT mice (C) mRNA for the myeloid lineage specific TRAP transcript 1C in adipose tissue of WT and TRAP+ mice. (D) Expression of monomeric TRAP in adipose tissue (T) and isolated adipocytes (Ad) from five subjects (Pat 1–5). (E) TRAP mRNA expression in human adipose tissue and in isolated adipocytes. (F) Co-localization between monomeric or total TRAP and the macrophage marker CD68 in subcutaneous human adipose tissue from a representative subject. (G) Monomeric TRAP is secreted from the mouse macrophage cell line RAW 264.7 (lane 1), whereas proteolytically processed TRAP is not detectable (lane 2).

In line with previous studies in animal models of obesity [21], [22], mRNA for c-fms (Figure 5A; Z = 2.72 p = 0.01) as well as F4/80 staining (Figure 5B) were increased in adipose tissue from obese TRAP+ mice indicative of an increase of infiltrating macrophages in adipose tissue. The mouse TRAP gene contains multiple promoters; the most proximal to the first coding exon contains a compound start site region used selectively by macrophages and osteoclasts [27]. A completely separate promoter upstream, which generates an alternative 5′UTR, is used by the hepatocytes and renal tubule cells, which are the other major sites of TRAP expression. The increased TRAP mRNA expression in TRAP+ adipose tissue (Figure 2E) probably originated from macrophages, since the myeloid lineage–specific TRAP 1C transcript [27] constituted 87% of TRAP mRNA transcripts in both TRAP+ and WT mice (Figure 5C). This is consistent with the analysis in the original description of this transgene [10] in which over-expression in macrophages and osteoclasts was directly demonstrated in tissue and isolated cells. Consistent with the high conservation of the TRAP promoter across species [27], in human adipose tissue, TRAP was mainly expressed by cells in the stroma cell fraction (TRAP mRNA in tissues vs cells; t = −2.88 p = 0.008 df = 26) (Figure 5D–E) identified by immunohistochemistry as CD68 positive macrophages (Figure 5F). Finally, we examined in vitro whether macrophages can secrete monomeric TRAP. The supernatant of the mouse macrophage cell line RAW 264.7 stimulated with LPS and IFN-γ was shown to contain exclusively monomeric TRAP (Figure 5G).

### TRAP overexpression affects adipokine- and cytokine profile in mouse adipose tissue and serum

**Figure 6:**
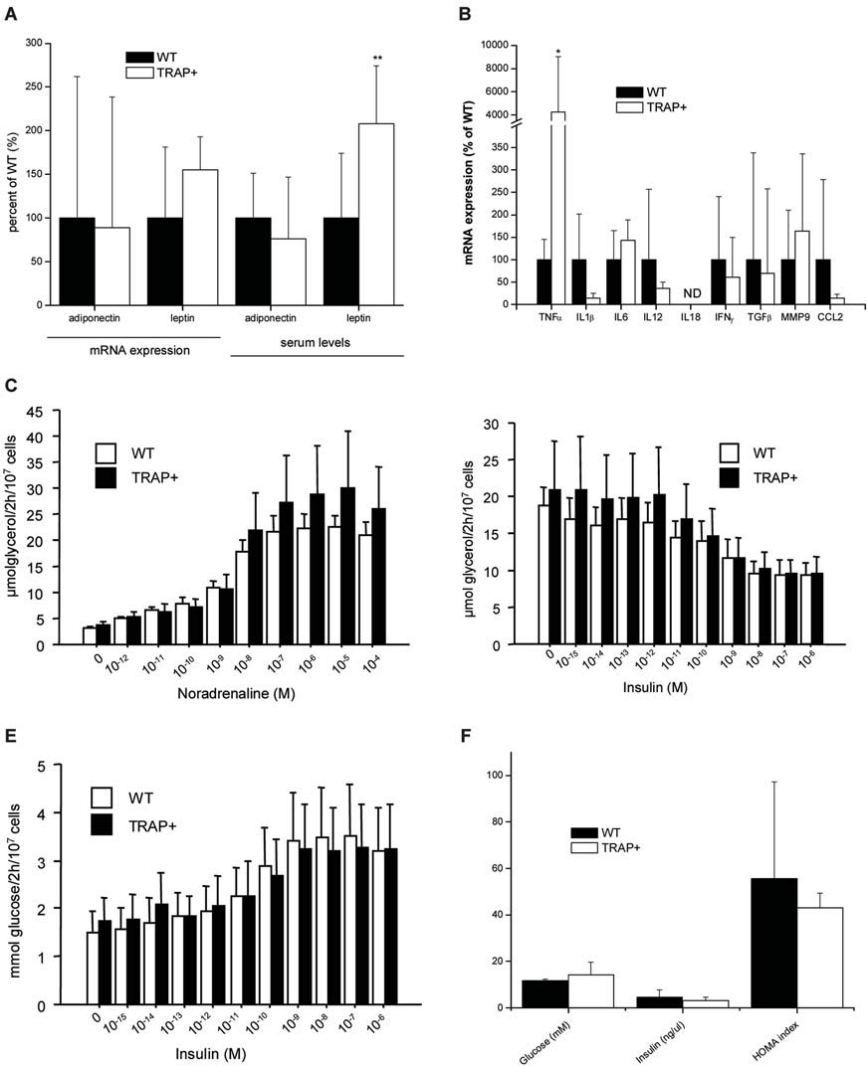
Metabolic and inflammatory profile of adipose tissue from obese TRAP over expressing mice. (A) Levels of leptin and adiponectin mRNA and serum protein in WT and TRAP+ mice. (B) Inflammatory gene expression profile in the TRAP+ mice. (C) Noradrenalin-stimulated lipolysis in isolated adipocytes from TRAP+ and WT mice. (D) Insulin-inhibitable lipolysis in isolated adipocytes from TRAP+ and WT mice. (E) Insulin- stimulated lipogenesis in isolated adipocytes from TRAP+ and WT mice. (F) Levels of blood glucose and insulin in TRAP+ mice.

Obesity in humans is associated with a so-called metabolic syndrome which involves alterations in circulating levels of a wide range of adipocyte and immune cell-derived cytokines, and eventually with insulin resistance. We measured mRNA and serum protein levels of cytokines and adipokines known to be altered in obesity in TRAP+ and WT mice. These factors can be divided into two groups: those only expressed by adipocytes (adiponectin, leptin) [28] and those thought to be expressed by several different cells in adipose tissue (TNFα, IL1β, MMP9, IL6, CCL2) [28] The mRNA levels of leptin and adiponectin in adipose tissue were found to be unchanged between the TRAP+ mice and WT littermates (Figure 6A). The serum leptin levels were two-fold increased (Figure 6A; Z = −2.83 p = 0.004) in the TRAP+ compared to WT mice, probably reflecting the overall increase in total body fat (and total fat cell number). The mRNA level of TNFα in adipose tissue was increased (Figure 6B; Z = 2.34 p = 0.019) in TRAP+ mice compared to WT littermates although serum TNFα levels were not (data not shown). The mRNA levels for CCL2, MMP9, IL1β, IFNγ, IL12b, TGFβ and IL6 were not significantly affected in the adipose tissue of TRAP+ mice (Figure 6B), suggesting that there is not a global activation of macrophages to produce inflammatory cytokines.

### Transgenic mice over expressing TRAP have normal adipocyte lipid metabolism and insulin sensitivity

Adipocytes isolated from TRAP+ mice exhibited normal spontaneous basal lipolysis (Figure 6C, D), normal responses to addition of noradrenaline (Figure 6C) and insulin (Figure 6D) on lipolysis and unchanged basal or maximum insulin induced lipogenesis (Figure 6E). Half-maximum effective concentrations of insulin and noradrenaline to elicit metabolic responses were also similar in adipocytes from WT and TRAP+ mice (data not shown). Thus, adipocyte metabolism and insulin sensitivity were normal in TRAP+ mice. In line with the finding in adipocytes, serum levels of glucose and insulin as well as HOMA index did not differ between the TRAP+ mice and their non-transgenic WT controls (Figure 6F).

## Discussion

TRAP overexpressing mice were originally generated in order to study the skeletal phenotype and bone turnover [10]. Unexpectedly, in the course of breeding to a different genetic background we found one sub-strain of TRAP over expressing mice that presented an early onset obesity that did not seem to be due to apparent over-eating. The differences in body weight (∼20–40%) was evident already at one month of age and up to 1 year of age the body weight curves in TRAP+ and WT mice were parallel. This indicates that other features such as decreased physical activity/energy expenditure could explain the obesity in TRAP+ animals. It is of interest to note that there was clear relative difference in organ size of TRAP+ animals, where most internal organs were relatively smaller in the TRAP+ mice compared to WT mice. This might suggest that adipose tissue expanded in TRAP+ mice leading to redistribution of energy expenditure on behalf of other organs which diminished in size.

In the TRAP over expressing mice, adipocyte volume was normal and several genes known to increase adipogenesis (PPARγ, GPDH and LPL) were up regulated. This suggested that the obesity was largely due to enhanced formation of new fat cells through differentiation and/or proliferation rather than lipid filling of pre-existing adipocytes, i.e. the TRAP+ mouse develops hyperplastic obesity. Only two previous models have demonstrated hyperplasia, rather than hypertrophy, as the major cause of obesity [29], [30]. The results also suggested that the monomeric form of TRAP is specifically causing the effect of TRAP on adipogenesis. A second sub-strain (TRAP+p) without detectable expression of monomeric TRAP, but with elevated levels of the proteolytically processed form in adipose tissue was lean.

Since the lines derive from the same founder, and over-express the mRNA and total protein to the same extent, we suggest that there is an independently segregating variant allele of a gene involved in the proteolytic processing of TRAP. Assuming this allele exists, it was acquired fortuitously in a complex breeding history from the original transgene, so the process clearly cannot be repeated precisely. This may pose a limitation to generate sublines of the desired phenotype, but since the original transgene construct gave very reproducible over-expression, new founders could be readily back-crossed to different inbred mouse strains to generate congenic strains in order to recapitulate the “obese” phenotype on more defined genetic backgrounds. We can assume that the corresponding “lean” allele could be identified genetically by performing a genome scan of a set of individual lean and fat individuals and a candidate genetic interval could be refined by intercrossing lean and fat sublines. It may also be that screening inbred mouse lines for a preponderance of monomeric TRAP could identify the genetic origin of the allele. Finally, comparative array profiling of macrophages from the two sub lines could also contribute to identification of a presumptive candidate modifier gene that is specific to “lean” animals. Alternatively, generating transgenic mice using for instance the aP2 promoter to direct over expression of the TRAP gene selectively to adipocyte lineage cells may provide another viable strategy to obtain mice with the obese phenotype.

TRAP knock out mice [31] have not been reported to have an altered adipose tissue phenotype, although our work would suggest more detailed studies would be worthwhile. This does not necessarily imply that monomeric TRAP has no role in normal adipose development, since the role of TRAP in bone is also partly redundant (the knockout animals are only mildly osteopetrotic). An interesting feature of the TRAP-over expressing transgenic mice is that there is an increased macrophage content in adipose tissue, so TRAP is directly or indirectly proinflammatory. A systemic effect of TRAP on macrophages could also underlie the increase in relative spleen size in these animals. It may be that in adipose tissue, as in bone, there is a complex homeostatic interaction between TRAP-producing and TRAP-responsive cells. It is interesting to note that elevated levels of serum TRAP previously has been positively correlated with serum insulin levels in NIDDM rat models [32]. In osteoclasts the post-translational proteolytic processing of TRAP is partly involving the cysteine protease cathepsin K [33]. Cathepsin K has been shown to be a marker of adiposity [34] and cathepsin K null mice gained less weight when put on high-fat diet and exhibited increased adipocyte lipolysis [35]. However, while TRAP seems to be mainly expressed in macrophages, cathepsin K seems to be expressed in the adipocyte itself [36] suggesting distinct roles for these proteins in adipose tissue.

The clear relevance of the mouse model was demonstrated in human patient material, where an increased expression of monomeric TRAP, but not proteolytically processed TRAP, was apparent in adipose tissue of obese subjects. Human obesity is commonly characterized by a combination of hypertrophic and hyperplastic adipose tissue, although hypertrophy usually dominates [37], [38], but some subjects exhibit predominantly hyperplastic (i.e. smaller adipocyte volume than expected) obesity [39]. When our obese subjects were divided according to fat cell size both TRAP mRNA and monomeric TRAP were showed a tendency to a further increase in those with hyperplastic obesity as compared to those with hypertrophic obesity.

In both hMSC and pre-adipocytes from mouse and humans monomeric TRAP stimulated both proliferation and differentiation, measured as increase of GPDH enzyme activity. However, proteolytically processed TRAP had no effect on these processes. These data are somewhat unexpected, as usually a positive effect on proliferation is accompanied by a negative effect on differentiation and the opposite. Nevertheless, the findings are consistent with the hypothesis that monomeric TRAP could exert a dual effect on both proliferation and differentiation of pre-adipocytes which would increase the impact of monomeric TRAP although the effect was small in each step. It is, however, possible that the adipogenic effect of monomeric TRAP is stronger in vivo than in vitro. The effect on differentiation in vitro was observed in the presence or absence of roziglitazone suggesting that monomeric TRAP can activate adipogenesis at variable levels of PPARγ activity. Although TRAP has not been shown previously to affect the proliferation and differentiation of adipocytes, TRAP has been shown to induce differentiation of osteoblasts [9] and haematopoietic cells [40].

Obesity is associated with a low-grade inflammation in adipose tissue [41], [42], where a hall-mark is influx of macrophages into the tissue [21], [22]. The latter has been hypothesized to be a consequence of adipocyte necrosis because of adipocyte hypertrophy [43] and/or increase of monocyte attractants such as MCP-1/CCL2 [44]–[46]. TRAP is normally expressed in cells from the myeloid linage [11], [47], [48] and since the construct in the transgenic TRAP over expressing mouse used in this study is under the influence of the normal TRAP promoter [10] we expected that only cells normally competent to express TRAP would have an elevated expression of the enzyme. Confirming this idea, we found that in mouse adipose tissue, almost 90% of the TRAP transcripts originated from the myeloid specific transcript 1C and in human adipose tissue, TRAP were mainly expressed in CD68 positive macrophages. This indicates that the main source of TRAP in adipose tissue is macrophages. It should be noted, however, that CD68 is expressed in cell types other than macrophages, e.g. fibroblasts, although the antibody used in this study has been shown to recognize CD68 preferably in macrophages [49].

It is unlikely that monomeric TRAP is secreted by other cells than macrophages since the 1C transcript, which is the predominant transcript in adipose tissue, is regulated by PU.1 and MiTF which are myeloid specific transcription factors [50]. Furthermore, the TRAP staining in the original transgene study [10] does not support the idea that significant levels of TRAP can be expressed by non myeloid cells other than in liver and kidney. However, we cannot rule out that secretion of TRAP from cells other than macrophages occur within adipose tissue.

In macrophages, TRAP has traditionally been considered a lysosomal constituent. However, recent studies in osteoclasts has shown that there is likely to be two populations of TRAP present, one intracellular which is mainly proteolytically processed and one secreted which is mainly monomeric [33]. Previously published data [16] as well as in vitro experiments of this study have shown that monomeric TRAP can be secreted also from macrophages in vitro. This is also in line with other studies showing that monomeric, rather than proteolytically processed TRAP seems to be the dominant secreted form also in other cell types [13], [51]. Together these data suggest that also in macrophages different isoforms of TRAP might be present intracellularly or secreted.

Once secreted from macrophages, monomeric TRAP seemingly acts on adipose tissue to induce adipogenesis. The molecular mechanism behind the action of monomeric TRAP on adipogenesis is unknown, but it can be speculated that TRAP is recognized by a surface receptor on pre-adipocytes and that signals from this receptor regulate adipogenesis.

Obesity is strongly linked to insulin resistance [52], [53] and several studies have highlighted the correlation between adipocyte size rather than adipose tissue mass and insulin malfunction [53]–[58]. The enlargement of adipocytes may not have pathophysiological significance by itself but rather be a manifestation of other pathogenetic factors leading to insulin resistance [58]–[60], such as increased adipocyte lipolysis resulting in elevated fatty acids, which in turn cause insulin resistance [61]. On the other hand, enlarged adipocytes may be pathogenic themselves by, for example, a change in production of adipokines and cytokines [58], the latter linking adipose inflammation to insulin resistance. Recently it was also shown that TNFα down regulates eNOS expression in metabolically active tissues which might sustain obesity [62]. Certain macrophage populations in adipose tissue might be associated with insulin resistance and obesity [26]. As judged by circulating insulin and glucose levels as well as HOMA index and by measurement of adipocyte lipolysis and lipogenesis, the obese TRAP+ mouse exhibited normal adipocyte lipid and glucose metabolism as well as insulin and catecholamine sensitivity, although it can not be excluded that some alterations of insulin action occurred in skeletal muscle or liver, which were not directly examined. Also, there was no evidence of major changes in expression of adipokines or cytokines involved in obesity mediated insulin resistance except for an increased mRNA expression of TNFα. Thus, the absence of fat cell hypertrophy and the modest change in expression of cytokines associated with an innate immune response may explain why obese TRAP+ mice had nor or little evidence of insulin resistance or altered fat cell metabolism in spite of macrophage infiltration. The enhanced number of adipocytes in the enlarged adipose tissue might cause the increased level of circulating leptin in TRAP+ mice.

In light of these data, we propose a hypothesis of a paracrine effect of monomeric TRAP on adipogenesis. In this model, monomeric TRAP, likely secreted predominantly by macrophages, influences the development of obesity by inducing adipogenesis in a situation where there is an influx of macrophages into the adipose tissue or an up regulation of monomeric TRAP in pre-existing macrophages. This could increase the formation of new, normally sized adipocytes with normal insulin sensitivity and metabolism. This hypothesis is not apparently consistent with the proposed function of macrophages in adipose tissue, since they previously have been reported to secrete factors that increases expression of inflammatory genes in adipocytes [23], [24] and inhibits adipogenesis [24], [25]. However, macrophages consist of a heterogeneous population of cells and can be activated differently [63] and evidence was recently presented for the presence of distinct macrophage populations in adipose tissue some being protective against insulin resistance [64]. With respect to expression of monomeric and proteolytically processed TRAP in macrophages it has been shown that monomeric TRAP has a more restricted distribution than proteolytically processed TRAP [11]. However, the regulation of the different forms of TRAP in macrophages remains to be elucidated. Thus, macrophages, depending on their progeny and/or activation state, might secrete factors, for example monomeric TRAP, which could have variable effects on adipose tissue.

In summary, certain macrophages in adipose tissue seem to secrete monomeric TRAP that induces obesity; at least partly due to adipogenic affects causing increased formation of normally sized adipocytes with normal insulin sensitivity, lipid and carbohydrate metabolism and adipokine/chemokine production.

## Materials and Methods

### Experimental animals

Age-matched male and female mice from different litters (WT , TRAP+p and TRAP+) were kept under controlled light/dark conditions with food and water available ad libitum. The study was approved by the Stockholm South Animal Ethical Committee (S159/01 and S235/04) and maintained according to the guidelines of the Animal Welfare Board at Karolinska Institutet.

### Statistical analysis

For statistical method and number of animals or subjects for each experiment see specific analysis. Values are given as mean±SD. Symbols for statistical comparison are * p<0.05; **p<0.01; ***p<0.005. NS; not significant

### Generation and genotyping of TRAP over expressing transgenic FVB/N mice

TRAP over expressing transgenic FVB/N mice [10] (TRAP+ or TRAP+p) containing >30 copies of the TRAP gene were used. Genomic DNA was purified using Puregene (Gentra, Minneapolis, MN) from mouse-tails. Primers (Invitrogen, Carlsbad, CA)/probes (Biosearch Technologies, Novato, CA) for SV40 and TRAP were as follows; (A) SV40 primers 5′CACCTGGTTGCTG ACTAAT TGAGA 3′/5′GTGTGTCAGTTAGGGTGTGGAAA 3′ and HEX labeled probe 5′CCCCAGGCTCCCCAGCAGGCAG 3′ annealing temperature 62°C. (B) TRAP primers 5′ TGGTCATTTCTTTGGGG CTTATCT 3′/5′GCTACTTGCGGTTTCACTATGGA 3′ and FAM labeled probe 5′TGTGA AGCCGCCCAGGGAGTCCTC 3′ annealing temperature 62°C. qPCR was run as stated under “Total RNA purification and RT-qPCR” with the exception that iQ Supermix (Bio-Rad, Hercules, CA) was used.

### Determination of organ weight index

Organs were dissected out and the wet weights were determined. For n see Table S2. Statistical analysis was carried out using Mann-Whitney U test.

### Determination of body weight–growth curves

Male (WT; n = 50, TRAP+p; n = 21, TRAP+; n = 56; for n at specific ages see Table S3) and female (WT; n = 68, TRAP+p; n = 22, TRAP+; n = 52; for n at specific ages see Table S3) were weighed at different time points. Statistical analyses were carried out using Kruskal Wallis followed by Mann Whitney U test.

### Measurement of lean mass and body fat content using DXA

Dual-energy X-ray absorptiometry (DXA) was performed as described [65]. Male (WT; n = 24, TRAP+p; n = 17, TRAP+; n = 5) and female (WT; n = 28, TRAP+p; n = 14 , TRAP+; n = 9 ), 2–12 month mice were used (Supplementary Table IV). Statistical analysis was carried out using Kruskal Wallis test followed by Mann-Whitney U test.

### Calculation of adipocyte volume

Adipocyte size was determined as described in [66]. Adipose tissue was obtained from the animals (WT; n = 8, TRAP+; n = 8) and adipocytes were isolated by collagenase treatment. Using direct microscopy, the diameter of 100 cells was determined and the mean fat cell volume was calculated. Statistical analysis was carried out using ANOVA.

### Measurement of food intake

Animals were given a specific amount of food and the consumption was measured by weighing the remaining food every other day for two weeks. Food consumption was then calculated as intake of gram food/gram body weight over two weeks for male (WT; n = 3, TRAP+; n = 6) and female (WT; n = 3, TRAP+; n = 5) mice. Statistical analysis was carried out using Mann-Whitney U test.

### Total RNA purification and RT-qPCR on mouse and human adipose tissue

Total RNA was extracted from gonadal or mesenteric adipose tissue of male and female mice (WT; n = 11, TRAP+p; n = 7, TRAP+; n = 9) using RNeasy Lipid Tissue Mini Kit (QIAGEN, Hilden, Germany), treated with DNase (Invitrogen, Carlsbad, CA), quantified using Ribogreen (Invitrogen, Carlsbad, CA) and then transcribed using iScript cDNA Synthesis Kit (Bio-Rad, Hercules, CA). From human subjects, total RNA was extracted from 300 mg subcutaneous fat tissue (n = 28) using RNeasy Mini Kit (QIAGEN, Hilden, Germany), determination of RNA purity and reverse transcription was performed as described [67]. Real-Time PCR was carried out on an iCycler iQ Real Time PCR Detection System (Bio-Rad, Hercules, CA) in triplets using, for mouse cDNA, Platinum® SYBR® Green qPCR SuperMix UDG (Invitrogen, Carlsbad, CA) and, for human cDNA, iQ SYBR Green Supermix (Bio-Rad, Hercules, CA) both with the addition of 10 nM fluorescein (Bio-Rad, Hercules, CA) in a final volume of 25 µl. Program setting for mouse samples were as follows: 2 min at 50°C, 2 min at 95°C, 40 cycles of 15 sec at 95°C and 30 sec at 62–64°C (depending on primer pair). Program setting for human samples were as follows: 10 min at 95°C, 40 cycles of 15 sec at 95°C 20 sec at 63°C. For primer information see Table S1. Primer pairs were optimized prior to use for primer concentration and annealing temperature to achieve PCR amplification efficiency between 95–105%. Mouse samples were normalized towards β-actin and human samples towards GAPDH. Relative quantification of mRNA was calculated using the “Comparative Ct method” as described in User Bulletin 2 from Applied Biosystems. For mice, statistical analysis was carried out using Mann-Whitney U test and for humans using ANOVA (Table S5).

### Measurement of TRAP enzyme activity

TRAP enzyme activity was measured [68] in tissue homogenates prepared as follows. Adipose tissue from male and female mice (WT; n = 11, TRAP+; n = 10, TRAP+p; n = 10) was homogenized in 0.15 M KCl+0.1% Triton X-100+Pefabloc (10 mg/ml)+Complete, Protease Inhibitor Cocktail Tablets (1 tablet/50 ml solution) (Boehringer Mannheim, Mannheim, Germany) and centrifuged at 3200 × g for 30 minutes. The supernatant was then assayed for TRAP enzyme activity.

### Human studies

Human abdominal subcutaneous adipose tissue was obtained from different sources. For the isolation of pre-adipocytes and mesenchymal stem cells (MSC), tissue was obtained as the product of cosmetic liposuction on otherwise healthy non-obese women (body mass index ( = BMI): 22–36 kg/m^2^; age 22–56 years; n = 10). For immunohistochemistry, tissue was obtained during surgery from three obese subjects undergoing gastric banding. For the comparison of gene and protein expression in lean versus obese subjects a frozen (−70°C) 300 mg tissue piece was used. It was obtained by needle biopsy from lean (BMI<25 kg/m^2^) or obese (BMI>30 kg/m^2^) but otherwise healthy women participating in ongoing studies of the genetic regulation of human fat cell function. For those in the gene expression study age (mean±SD) was 36±14 and 39±7 years in lean (n = 14) and obese (n = 14) subjects, respectively. BMI was 23±2 and 36±4 kg/m^2^ respectively. Fat cell volume was 450 ±140 and 822±240 pL in lean and obese, respectively. The obese group in the gene expression study was sub-divided in two groups according to fat cell size; hyperplasia (fat cell volume of 641±450 pL; n = 7) and hypertrophia (cell volume of 1004±490 pL; n = 7) with no between group difference in BMI. In the protein expression study age was 40±4 years (lean (n = 7)) and 40±13 years (obese (n = 12)), the BMI was 23±1 kg/m^2^ and 38±5 kg/m^2^, and fat cell volume was 490±190 pL and 868±223 pL, respectively. The obese group was sub-divided into a hyperplastic (fat cell volume 659±420 pL; n = 6) and hypertrophic (fat cell volume 1077±570 pL; n = 6) group) with no between group difference in BMI. The human ethics committee at the Karolinska University Hospital Huddinge approved the study, which was explained in detail to each subject and written informed consent was obtained. Unpaired t-test was used to compare lean and obese subjects and adipose tissue versus adipocytes. ANOVA was used to compare lean subjects with the hypertrophic and hyperplastic groups. Degrees of freedom in these experiments were 1, 1 and 2, respectively.

### Electrophoresis and immunoblot analysis

Partially purified [11] culture media from 1×10^6^ RAW 264.7 cells or 70mU TRAP from WT/TRAP+p/TRAP+ adipose tissue was subjected to SDS-PAGE using 12% NuPage gels (Invitrogen, Carlsbad, CA) run with MOPS buffer and transferred to PVDF membranes (Bio-Rad, Hercules, CA). Membranes were blocked using 1% TBST (100 mM Tris-HCl pH 7.6, 154 mM NaCl, 1% Tween-20) and stained with rabbit anti-mouse monomeric TRAP [11] 1∶1500 or rabbit anti-rat total TRAP 1∶1500 [69], goat anti-rabbit HRP 1∶10 000 (Calbiochem, La Jolla, CA) and developed using Renaissance (NEN Life Science, Boston, MA). Human samples; 100 µg of total protein total obtained from protein lysates was separated on 12% PAA-gels, transferred onto PVDF membranes and stained using rabbit anti-mouse monomeric TRAP or rabbit anti-rat total TRAP as described above. Bands were detected using Supersignal® (Pierce, Rockford, IL). Relative expression was determined using Chemidoc XRS System (Bio-Rad, Hercules, CA).

### Immunohistochemistry

Immunohistochemistry was carried out mainly as previously described [11]. Mouse adipose tissue from male and female mice; paraffin sections (WT; n = 3, TRAP+; n = 3) were treated with 0.1% trypsin (Sigma-Aldrich, St. Louis, MO) at 37°C for 30 minutes and stained for macrophages using F4/80 monoclonal antibody (1∶50) (Serotec, Oxford, UK). ChemMate Detection Kit Peroxidase/DAB rabbit/mouse (Dako, Glostrup, Denmark) was used as secondary antibodies and developing solution. Human adipose tissues; microwave treated (1mM EDTA pH 8 for 10 minutes at 900 W) paraffin sections were stained with rabbit anti-rat total TRAP 1:50 [69] recognizing both monomeric and the two-subunit TRAP or rabbit anti-mouse monomeric TRAP antibody 1∶50 [11] and CD68 1∶100 (Dako, Glostrup, Denmark). Secondary antibodies were ALEXA 568 goat anti-rabbit Fab_2_ 1∶250 and ALEXA 488 goat anti-mouse Fab_2_ fragments 1∶100 (Invitrogen, Carlsbad, CA). Sections were then examined using a Leica TCS NT ArKr laser confocal microscope (Leica Microsystems AG, Wetzler, Germany).

### Expression, purification and cleavage of recombinant rat TRAP

Recombinant rat TRAP was expressed in Sf9 insect cells and purified as described [70]. To generate the proteolytically processed form, recombinant rat TRAP was digested at 37°C for 40 minutes with recombinant human cathepsin K (kindly provided by Dr. Robert Dodds) in 5mM NaAc pH 5.5, 1 mM EDTA and 10 mM DTT using a 1∶1 molar ratio.

### Proliferation and differentiation of 3T3-L1 cells in the presence of TRAP

For proliferation experiments (n = 4, df = 3); 3T3-L1 (ATCC (LGC Promochem, Borås, Sweden)) cells (2,000 cells/cm^2^) were cultured in DMEM/F12 Glutamax II, 4.5 g/L glucose, 1.5 g/L sodium bicarbonate, penicillin/streptomycin, 2.5% calf bovine serum +/− cleaved (600 U/mg) or monomeric (50 U/mg) TRAP (10^−9^ M–10^−12^ M). After 24, 48 and 72 h, cells were labeled for 2 h with BrdU, fixed and BrdU incorporation was measured using Cell Proliferation ELISA, BrdU kit (Roche, Mannheim, Germany).

For differentiation experiments (n = 4, df = 3); 3T3-L1 cells (6,000 cells/cm^2^) were grown into confluence in DMEM/F12 Glutamax II, 4.5 g/L glucose, 1.5 g/L sodium bicarbonate, penicillin/streptomycin, 10% calf bovine serum +/− cleaved or monomeric TRAP (10^−9^ M–10^−12^ M)). At 2–3 days after confluence, 0.5 mM isobutylmethylxanthine, 1 µM dexamethasone and 10 µg/ml bovine insulin was added to the media to start differentiation. Cells were then cultured +/− roziglitazone (1 µM), cleaved (600 U/mg) or monomeric (50 U/mg) TRAP (10^−9^ M–10^−12^ M). After 48 h, dexamethasone, isobutylmethylxanthine and roziglitazone were omitted. After an additional 48h (i.e. 4 days after the start of differentiation), cells were lysed and GPDH activity was measured [71]. Statistical analysis was performed using t-test.

### Proliferation and differentiation of human mesenchymal stem cells derived from adipose tissue in presence of TRAP

Human MSC were isolated from a subcutaneous lipoaspirate, grown to 60–70% confluence and passage for at least 20 passages, as described [72]. For proliferation experiments (n = 3, df = 2); hMSC (passage 9) (2,000 cells/cm^2^) were cultured in DMEM Glutamax I, 1 g/L glucose, penicillin/streptomycin, 2.5% fetal bovine serum in the presence or absence of cleaved (600U/mg) or monomeric (50U/mg) TRAP (10^−9^ M–10^−12^ M). After 48 h, cells were labeled for 2h with BrdU, fixed and BrdU incorporation was measured as described above.

For differentiation experiments (n = 3, df = 2); hMSC (passage 18) were differentiated as described [72] in the presence or absence of cleaved or monomeric TRAP (10^−9^ M). Twelve days after the start of differentiation cells were lysed and GPDH activity was measured [71]. Statistical analysis was performed using t-test.

### Differentiation and proliferation of preadipocytes isolated from human adipose tissue in presence of TRAP

Isolation of pre-adipocytes were performed as previously described [73]. For proliferation experiments (n = 3, df = 2); cells (4000 cells/cm^2^) were cultured in growth medium containing 2.5% fetal bovine serum in the presence or absence of cleaved or monomeric TRAP (10^−9^ M–10^−12^ M). After 5 days, cells were labelled for 2 h with BrdU, fixed and BrdU incorporation was measured as described above. For differentiation experiments (n = 3, df = 2); cells were differentiated as described [74] in the presence or absence of cleaved (600U/mg) or monomeric (50 U/mg) TRAP (10^−9^ M–10^−12^ M). Twelve days after the start of differentiation, cells were lysed in GPDH buffer and GPDH activity was measured as described [71]. Statistical analysis was performed using t-test.

### Secretion of TRAP from macrophages

RAW 264.7 cells were cultured in a 37°C humidified 5% CO_2_ atmosphere in DMEM media (Gibco, St Louis, MO) supplemented with 10% FCS (Gibco, St Louis, MO) and 0.1 mg/ml gentamycin (Gibco, St Louis, MO). For stimulation, 0.25×10^6^ RAW 264.7 cells/ml (passage 3) was treated with IFNγ (500 U/ml) (Invitrogen, Carlsbad, CA) for 16 h., and then with LPS (1 ng/µl) (Sigma-Aldrich, St. Louis, MO) for an additional 24 h.

### Metabolic studies on the TRAP over expressing mice

Lipolysis and lipogenesis experiments were conducted on isolated adipocytes as described [73] on fat (pooled fractions of gonadal, mesenteric and inguinal fat from the same animal) from male and female mice (WT; n = 8 and TRAP+; n = 8). After a 2 h incubation, the cell suspension was subjected to measurement of active uptake into total fat cell lipids (lipogenesis index). Statistical analysis was performed using ANOVA.

### Measurement of glucose, insulin and HOMA index

Glucose (WT; n = 3 and TRAP+; n = 3), HOMA index (WT; n = 3 and TRAP+; n = 3) and insulin (WT; n = 8 and TRAP+; n = 3) were determined individually on serum from male and female mice, using a Monarch automated analyzer (ILS Laboratories Scandinavia AB, Sollentuna, Sweden) or a RIA assay (Linco Research Inc., St. Charles, MO), respectively. Statistical analysis was performed using Mann-Whitney U test.

### Measurement of serum leptin, adiponectin and TNFα

Serum levels of leptin in male and female mice >4 months of age (WT; n = 26 and TRAP+; n = 13) (Quantikine Mouse Leptin Immunoassay, R&D Systems, Inc., Minneapolis, MN), of adiponectin in male and female mice >4 months of age (WT; n = 11 and TRAP+; n = 7) (Adiponectin Mouse ELISA, BioVendor, Heidelberg, Germany) and of TNFα in male and female mice >4 months of age (WT; n = 8 and TRAP+; n = 5) (Ready-Set-Go! Mouse TNFα ELISA, ebioscience, San Diego, CA) was measured. Statistical analysis was carried out using Mann-Whitney U test.

## Supporting Information

Table S1 (0.05 MB DOC)

Table S2 (0.03 MB DOC)

Table S3 (0.06 MB DOC)

Table S4 (0.04 MB DOC)

Table S5 (0.03 MB DOC)

Table S6 (0.05 MB DOC)
