# Supplementary material for: Monomeric Tartrate Resistant Acid Phosphatase Induces Insulin Sensitive Obesity
Source: PLoS One. 2008 Mar 5;3(3):e1713. doi: 10.1371/journal.pone.0001713 (PMC2248616; doi:10.1371/journal.pone.0001713)
Supplement: Table S1 — (0.05 MB DOC) [file pone.0001713.s001.doc]

| **Table S1. Primer information for** | | **real time qPCR.** |  |  |  |
| --- | --- | --- | --- | --- | --- |
|  |  |  |  |  |  |
| **Gene/mRNA transcript** | **Species** | **Sense** | **Antisense** | **Primer conc. (nM)** | **Annealing temp.** |
| GAPDH | human | TCCACCACCC TGTTGCTGTAG | ACCCACTCCTCCACCTTTGA | 300/300 | 63 |
| TRAP | human | CGCACAGGTAGGCAGTGAC | CTACCCCGTGTGGTCCATAG | 300/300 | 63 |
| actin | mouse | AAGACCTCTATGCCAACACAGTG | CAGGAGGAGCAATGATCTTGATCT | 900/300 | 62 |
| adiponectin | mouse | CTAAGGGTGAGACAGGAGATGTTG | AGCGATACATATAAGCGGCTTCTC | 900/900 | 57 |
| CCL2 | mouse | CCAGCCAACTCTCACTGAAG | ATGAGTAGCAGCAGGTGAGT | 300/300 | 55 |
| c-fms | mouse | TTTCTTGTGGTCAGGGTGCTTC | GCCCAGAACTGGTTGTAGAGC | 900/900 | 64 |
| F4/80 | mouse | CTTTGGCTATGGGCTTCCAGTC | GCAAGGAGGACAGAGTTTATGGTG | 900/900 | 60 |
| Fabp4 | mouse | CACCGAGATTTCCTTCAAACTG | GTCACGCCTTTCATAACACATT | 900/300 | 55.8 |
| GPDH | mouse | ATCAGGCTTGTCCAGGATTATG | TCACCTCTGCTTCAATGTATGG | 300/900 | 55.8 |
| HSL | mouse | AAGGAGAGGACAGCAAGGTA | CTCGTGGGATTTAGAGGTCTG | 900/300 | 55.8 |
| IFNγ | mouse | CACACTCATTGAAAGCCTAGAAAGTC | GAGGTAGAAAGAGATAATCTGGCTCTC | 300/900 | 56 |
| IL12 | mouse | CTTCTTCATCAGGGACATCATCAAAC | CTCCTCTGTCTCCTTCGTCTTTTC | 300/300 | 59 |
| IL18 | mouse | GACAACACACTTTACCTTATACCTGAAG | AACGAAGAGAACTTGGTCATTTATGC | 900/900 | 61 |
| IL1β | mouse | GAGCTTCAGGCAGGCAGTATC | ACCAGCAGGTTATCATCATCATCC | 900/900 | 56 |
| IL6 | mouse | ACTTCCATCCAGTTGCCTTCTTG | TCTCATTTCCACGATTTCCCAGAG | 900/900 | 59 |
| leptin | mouse | ACCATTGTCACCAGGATCAATGAC | GACCTGTTGATAGACTGCCAGAG | 900/900 | 57 |
| MMP9 | mouse | CACAGCCAACTATGACCAGGATAA | GCTTGCCCAGGAAGACGAAG | 900/900 | 61 |
| LPL | mouse | GAATGGCAAGCAACACAACC | GGAGTCGCTCATCCACTTAAG | 900/300 | 58.7 |
| PPARγ | mouse | ATGCTGTTATGGGTGAAACTCT | CAAAGGAATGCGAGTGGTCT | 300/900 | 57 |
| pref-1 | mouse | CCCTCTGTGACAAGTGTGTAA | GAGCATTCGTACTGGCCTTT | 300/50 | 55.8 |
| TNFα | mouse | GACCCTCACACTCAGATCATCTTC | CTCCGCTTGGTGGTTTGCTAC | 900/900 | 55 |
| TRAP | mouse | GCTACTTGCGGTTTCACTATGGA | TGGTCATTTCTTTGGGGCTTATCT | 900/300 | 62 |
| TRAP 1A transcript | mouse | GGTCAGGAGTGGGAGCCATAT | AAGAGCCTTCAAGTAAGTGGAACA | 900/900 | 60 |
| TRAP 1B transcript | mouse | TCCGCAGCTCAGTTGGGTAG | GCCCACAGCCACAAATCTCAG | 300/300 | 59 |
| TRAP 1C transcript | mouse | CTCTGACCACCTGTGCTTCCT | CTGTGTGGAATGGGGCATTGG | 900/900 | 65 |
