# Supplementary material for: Monomeric Tartrate Resistant Acid Phosphatase Induces Insulin Sensitive Obesity
Source: PLoS One. 2008 Mar 5;3(3):e1713. doi: 10.1371/journal.pone.0001713 (PMC2248616; doi:10.1371/journal.pone.0001713)
Supplement: Table S2 — (0.03 MB DOC) [file pone.0001713.s002.doc]

| **Table S2. Statistics for organ weight in WT and TRAP+ mice.** | | | | |  |
| --- | --- | --- | --- | --- | --- |
| Statistical data for Figure 1B. Statistical analysis was carried out using Mann-Whitney U test. | | | | |  |
|  |  |  | **Valid N** | |  |
| **Variable** | **Z value** | **p value** | **WT** | **TRAP+** |  |
| Heart | 2.54899 | 0.010804 | 11 | 5 |  |
| Kidney | 2.59238 | 0.009532 | 24 | 6 |  |
| Liver | 2.37384 | 0.017605 | 30 | 10 |  |
| Spleen | -2.48485 | 0.012961 | 23 | 11 |  |
| Brown fat | -2.13201 | 0.033007 | 4 | 6 |  |
| Mesenteric fat | -2.85774 | 0.004267 | 9 | 10 |  |
