# Supplementary material for: Monomeric Tartrate Resistant Acid Phosphatase Induces Insulin Sensitive Obesity
Source: PLoS One. 2008 Mar 5;3(3):e1713. doi: 10.1371/journal.pone.0001713 (PMC2248616; doi:10.1371/journal.pone.0001713)
Supplement: Table S3 — (0.06 MB DOC) [file pone.0001713.s003.doc]

| **Table S3. Statistics on growth curves of WT, TRAP+p and TRAP+ mice.** | | | | |  |  |  | |
| --- | --- | --- | --- | --- | --- | --- | --- | --- |
| Statistical data for Figure 2A and B. Statistical analyses were performed using Kruskal Wallis followed by Mann-Whitney U test | | | | |  |  |  | |
|  |  |  |  |  |  |  |  | |
|  |  | **n** | | **Kruskal Wallis** | | **Mann-Whitney** | | |
| **Genotype** | **Age (months)** | **male** | **female** | **male** | **female** | **male** | | **female** |
| WT | 1 | 6 | 7 |  |  |  | |  |
| WT | 2 | 4 | 11 |  |  |  | |  |
| WT | 3 | 20 | 22 |  |  |  | |  |
| WT | 6 | 1 | 5 |  |  |  | |  |
| WT | 7 | 7 | 11 |  |  |  | |  |
| WT | 8 | 6 | 4 |  |  |  | |  |
| WT | 11 | 6 | 8 |  |  |  | |  |
| TRAP+p | 1 | 5 | 5 | NS | NS | NS | | NS |
| TRAP+p | 3 | 9 | 9 | NS | Z = 3.20 p= 0.004 | NS | | Z = -3.83 p= 0.0001 |
| TRAP+p | 8 | 5 | 5 | NS | NS | NS | | NS |
| TRAP+p | 11 | 2 | 1 | NS | NS | NS | | NS |
| TRAP+ | 1 | 9 | 9 | Z = 2.89 p= 0.012 | Z = 3.79 p= 0.0004 | Z = -3.18 p= 0.001 | | Z = -3.33 p= 0.0009 |
| TRAP+ | 2 | 9 | 9 | NS | Z = 3.30 p= 0.0009 | NS | | Z = -3.30 p= 0.0009 |
| TRAP+ | 3 | 21 | 8 | Z = 5.57 p= 0.000000 | Z = 4.83 p= 0.000004 | Z = -5.24 p= 0.000000 | | Z = -4.13 p= 0.00004 |
| TRAP+ | 6 | 2 | 2 | NS | NS | NS | | NS |
| TRAP+ | 7 | 10 | 9 | Z = 3.42 p= 0.0006 | Z = 3.76 p= 0.0002 | Z = -3.41 p= 0.0006 | | Z = -3.76 p= 0.0002 |
| TRAP+ | 8 | 1 | 1 | NS | NS | NS | | NS |
| TRAP+ | 11 | 7 | 14 | Z = 3.04 p= 0.007 | Z = 3.74 p= 0.0006 | Z = -2.86 p= 0.004 | | Z = -3.75 p= 0.0002 |
|  |  |  |  |  |  |  | |  |
| Comparisons made are TRAP+p (WT vs TRAP+p) and TRAP+ (WT vs TRAP+). | | | |  |  |  | |  |
|  | |  |  |  |  |  | |  |
