# Supplementary material for: Monomeric Tartrate Resistant Acid Phosphatase Induces Insulin Sensitive Obesity
Source: PLoS One. 2008 Mar 5;3(3):e1713. doi: 10.1371/journal.pone.0001713 (PMC2248616; doi:10.1371/journal.pone.0001713)
Supplement: Table S4 — (0.04 MB DOC) [file pone.0001713.s004.doc]

| **Table S4. Statistics on relative lean and fat mass.** | | | |  |  |  |
| --- | --- | --- | --- | --- | --- | --- |
| Statistical data for Figure 2C and D. Statistical analysis was performed using Kruskal-Wallis test followed by Mann-Whitney U test. | | | | | | |
|  |  |  |  |  |  |  |
| **A) Male** |  |  |  |  |  |  |
|  | **Relative lean mass** | | **Relative fat mass** | |  |  |
|  | **WT vs TRAP+** | **TRAP+p vs TRAP+** | **WT vs TRAP+** | **TRAP+p vs TRAP+** |  |  |
| **Z** | 3.17543 | 3.25136 | -3.00222 | -3.32971 |  |  |
| **p-level** | 0.001496 | 0.001149 | 0.002680 | 0.000869 |  |  |
|  |  |  |  |  |  |  |
| **B) Female** |  |  |  |  |  |  |
|  | **Relative lean mass** | | **Relative fat mass** | |  |  |
|  | **WT vs TRAP+** | **TRAP+p vs TRAP+** | **WT vs TRAP+** | **TRAP+p vs TRAP+** |  |  |
| **Z** | 3.646160 | NS | -3.30986 | NS |  |  |
| **p-level** | 0.000266 | NS | 0.000932 | NS |  |  |
|  |  |  |  |  |  |  |
| NS = not significant | |  |  |  |  |  |
