# Supplementary material for: Monomeric Tartrate Resistant Acid Phosphatase Induces Insulin Sensitive Obesity
Source: PLoS One. 2008 Mar 5;3(3):e1713. doi: 10.1371/journal.pone.0001713 (PMC2248616; doi:10.1371/journal.pone.0001713)
Supplement: Table S5 — (0.03 MB DOC) [file pone.0001713.s005.doc]

| **Table S5. Statistics on mRNA and protein expression in human adipose tissue.** | | | |
| --- | --- | --- | --- |
| Statistical data for Fig. 3. Statistical analysis was performed using ANOVA and unpaired t-test followed by Bonferroni correction. | | |  |
|  |  |  |  |
| **Comparison** | **t or F value** | **p value** |  |
| lean vs obese, mRNA | 2.84 | 0.0090 |  |
| lean vs obese, protein | 2.77 | 0.0132 |  |
| lean vs hypertrophic obese, mRNA | 6.86 | 0.0040 |  |
| lean vs hyperplastic obese, mRNA | 3.38 | 0.0060 |  |
| lean vs hypertrophic obese, protein | 3.02 | 0.0200 |  |
| lean vs hyperplastic obese, protein | 2.70 | 0.0400 |  |
| ANOVA, mRNA | 6.86 | 0.0040 |  |
| ANOVA, protein | 5.39 | 0.0160 |  |
