# Supplementary material for: Monomeric Tartrate Resistant Acid Phosphatase Induces Insulin Sensitive Obesity
Source: PLoS One. 2008 Mar 5;3(3):e1713. doi: 10.1371/journal.pone.0001713 (PMC2248616; doi:10.1371/journal.pone.0001713)
Supplement: Table S6 — (0.05 MB DOC) [file pone.0001713.s006.doc]

| **Table S6. Statistics on in vitro proliferation and differentiation in the presence of different forms of TRAP.** | | | | | | | | | | | |  | |  |
| --- | --- | --- | --- | --- | --- | --- | --- | --- | --- | --- | --- | --- | --- | --- |
| Statistical data for Figure 4. Statistical analysis was performed using t-test. | | | | | |  |  | |  | | |  | |  |
|  |  | | |  | |  |  | |  | | |  | |  |
|  | **Proliferation monomeric TRAP (M)** | | | | | | **Differentiation monomeric TRAP (M)** | | | | | |  | |
| **Day** | **10^-9^** | | **10^-10^** | | **10^-12^** | | **10^-9^** | **10^-10^** | | **10^-11^** | **10^-12^** | |  | |
| **1** |  | |  | | t = 11.72 p = 0.007* | |  | t = 2.49 p = 0.04* | | t = 3.26 p = 0.02* | t = 2.88 p = 0.03* | |  | |
| **2** | t = 8.46 p = 0.01* | |  | |  | |  |  | |  |  | |  | |
| **2** | t = 4.32 p = 0.04** | | t = 7.58 p = 0.02** | | t = 8.00 p = 0.04** | |  |  | |  |  | |  | |
| **3** |  | |  | | t = 5.75 p = 0.03* | |  |  | |  |  | |  | |
| **4** |  | |  | |  | |  |  | |  |  | |  | |
| **5** | t = -12.74 p = 0.006*** | |  | | t = -3.41 p = 0.001*** | |  |  | |  |  | |  | |
| **12** |  | |  | |  | | t = 9.14 p =0.0003** |  | |  |  | |  | |
| **12** |  | |  | |  | | t = -38.90 p =0.0007*** | t = -5.33 p = 0.03*** | |  |  | |  | |
|  |  | |  | |  | |  |  | |  |  | |  | |
| *3T3-L1 | | |  | |  | |  |  | |  |  | |  | |
| ** human MSC | | |  | |  | |  |  | |  |  | |  | |
| *** human pre-adipocytes | |  |  | |  | |  |  | |  |  | |  | |
|  | | | | |  | |  |  | |  |  | |  | |
